# Energetic Changes Caused by Antigenic Module Insertion in a Virus-Like Particle Revealed by Experiment and Molecular Dynamics Simulations

**DOI:** 10.1371/journal.pone.0107313

**Published:** 2014-09-12

**Authors:** Lin Zhang, Ronghong Tang, Shu Bai, Natalie K. Connors, Linda H. L. Lua, Yap P. Chuan, Anton P. J. Middelberg, Yan Sun

**Affiliations:** 1 Department of Biochemical Engineering and Key Laboratory of Systems Bioengineering of the Ministry of Education, School of Chemical Engineering and Technology, Tianjin University, Tianjin, People's Republic of China; 2 Collaborative Innovation Center of Chemical Science and Engineering (Tianjin), Tianjin, People's Republic of China; 3 Australian Institute for Bioengineering and Nanotechnology, The University of Queensland, Brisbane, QLD, Australia; 4 Protein Expression Facility, The University of Queensland, Brisbane, QLD, Australia; University of Akron, United States of America

## Abstract

The success of recombinant virus-like particles (VLPs) for human papillomavirus and hepatitis B demonstrates the potential of VLPs as safe and efficacious vaccines. With new modular designs emerging, the effects of antigen module insertion on the self-assembly and structural integrity of VLPs should be clarified so as to better enabling improved design. Previous work has revealed insights into the molecular energetics of a VLP subunit, capsomere, comparing energetics within various solution conditions known to drive or inhibit self-assembly. In the present study, molecular dynamics (MD) simulations coupled with the molecular mechanics-Poisson-Boltzmann surface area (MM-PBSA) method were performed to examine the molecular interactions and energetics in a modular capsomere of a murine polyomavirus (MPV) VLP designed to protect against influenza. Insertion of an influenza antigenic module is found to lower the binding energy within the capsomere, and a more active state is observed in Assembly Buffer as compared with that in Stabilization Buffer, which has been experimentally validated through measurements using differential scanning calorimetry. Further in-depth analysis based on free-energy decomposition indicates that destabilized binding can be attributed to electrostatic interaction induced by the chosen antigen module. These results provide molecular insights into the conformational stability of capsomeres and their abilities to be exploited for antigen presentation, and are expected to be beneficial for the biomolecular engineering of VLP vaccines.

## Introduction

Virus-like particles (VLPs) are highly organized nanoparticles that self-assemble from viral structural proteins [1], [2], [3], and show great potential in vaccinology, gene therapy, drug delivery and materials science. The success of VLP-based vaccines for human papillomavirus [4], [5] and hepatitis B [6] demonstrates the potential of VLPs as safe and efficacious vaccines. Increasingly, research is directed to engineering the surface of modular VLPs to enhance immunogenicity of selected antigens. However, effect of inserting an antigen module on the self-assembly of VLP must be understood at a more fundamental level than is presently possible. Efficient antigen display on a VLP depends on the tolerance of the protein assembly and stability to modular insertions into surface exposed loops. Previous attempts to engineer surface loops of VLPs has led to destabilization of the structure such that assembly was no longer achieved [7], and further insertion of foreign peptides into some modular VLPs demonstrated a poor tolerance to such modifications, leading to mis-folded, aggregated or degraded proteins [8], [9]. Nevertheless, some insertions are well tolerated, with little to no effect on assembly [10], [11], [12], meaning the design process remains highly empirical. Joshi et al. [13] proposed a computer-aided vaccine design strategy to accelerate the discovery of vaccines with high immunogenicity and thermal stability. However, the disturbance on the self-assembly of VLPs induced by antigen module insertion is still a significant problem, especially at the molecular level. Facilitated by advanced investigations such as molecular dynamics (MD) simulations [14], [15], [16], [17], [18], [19], [20], [21], [22], further examination on antigen insertion tolerance for modular VLP assembly is required to speed advances in modular VLP vaccine platforms.

In our earlier work [23], the molecular interactions in the capsomere of a murine polyomavirus (MPV) VLP has been extensively investigated. In the present study, the molecular energetics within a modular capsomere of MPV VLP designed to protect against influenza was investigated using all-atom (AA) MD simulations coupled with the molecular mechanics-Poisson-Boltzmann surface area (MM-PBSA) method [24],[25],[26]. The energetic changes due to antigenic module insertion were examined with emphasis on their implications in structural regulation of VLP assembly. Further experimental validation of simulations was performed via VLP assembly characterization using Asymetrical Flow-Field-Flow Fractionation (AF4) and thermal stability was experimentally probed using differential scanning calorimetry (DSC).

## Models and Methods

### Model Construction

The system constructed is of a single modular capsomere as reported previously [23], with an additional influenza epitope module (VP1-GCN4-H190-GCN4 capsomere) as studied previously [27] denoted here as Ag-Cap, where H190 (STSADQQSLYQNADAY) is from H1N1 A/California/07/2009 influenza. The AA model of Ag-Cap was constructed using Accelrys Discovery Studio 3.0 (Accelrys, Inc., San Diego, USA), which is convenient for homology modeling. Herein, the ‘Build Homology Models' module in MODELLER program is used for the construction of Ag-Cap based on the structure of VP1 (PDB ID: 1SID) and influenza epitope with linkers (PDB ID: 3MLH and 2WPZ) as previously reported [27], and as shown in yellow in Fig. S1 in Supporting Information. Two solution conditions including Stabilization Buffer (S3) and Assembly Buffer (S4) were considered (Table 1) [23]. The simple point charge (SPC) model is used for water molecules. Na^+^ and Cl^-^ are considered as charged beads. For NH_4_^+^ and SO_4_^2-^, the topology was taken from the GROMOS96 43a1 force field. Each molecular system was first solvated in a cubic box (13×13×13 nm^3^). Solvent molecules were then added randomly around the protein, followed by the neutralization of the system by adding Na^+^ or Cl^-^ as counter ions. The numbers of ions and water molecules for each system are summarized in Table S1.

**Table 1:** Properties of Molecular Systems Used in MD Simulations.

Protein	System	Solution^a^	SS bonds^b^	Remark
Ag-Cap	S3	200 mM NaCl	-	Stabilization Buffer
	S4	0.5 M (NH_4_)_2_SO_4_, 1 mM CaCl_2_	+	Assembly Buffer

a The same solution conditions of S3 and S4 used for Cap reported previously [23] were used herein for Ag-Cap to examine the energetic changes due to antigenic module insertion.

b - and + represent absence and existence of disulfide bonds, respectively.

### Molecular Dynamics Simulations

MD simulations in the NPT ensemble were performed using GROMACS 4.5.3 package [28] (http://www.gromacs.org/) with the GROMOS96 43a1 force field, following the procedure proposed in the previous work [23]. Temperature was controlled at 310.15 K by the velocity-rescale (v-rescale) [29] method with a time constant of 0.5 ps. Pressure was controlled at 1 atm by the Berendsen barostat with coupling constants of 1.0 ps. Linear Constraint Solver (LINCS) algorithm [30] was applied to constrain all bonds, i.e., bonds were converted to constraints using the LINCS algorithm rather than represented by harmonic potential. LINCS algorithm [30] can reset bonds to their correct lengths after an unconstrained update. It is inherently stable and has been extensively used [31],[32],[33],[34],[35]. Periodic boundary was used in x, y, and z directions. Particle-mesh Ewald (PME) algorithm [36] was used to deal with the electrostatic interactions. Cutoffs of neighbor atom list, Coulomb potential, and Lennard-Jones (LJ) potential energies were all set to 0.9 nm. Initial velocities of particles were generated according to a Maxwell distribution. Verlet algorithm was used for integration with a time step of 2 fs. Prior to simulation, each system was subjected to 5000 steps of steepest descent energy minimization without limitations on the molecule, followed by 500 ps temperature and pressure equilibration process with position restraints on the protein heavy atoms. Then MD simulation for production dynamics was run for 20 ns as described above. Three independent simulations were performed for each set of condition.

### Conformation and Molecular Interaction Analysis

Simulation trajectories were analyzed using several auxiliary programs provided within GROMACS, including g_rms for the root-mean-square deviation of Cα (Cα RMSD) of the protein with respect to the initial structure, g_gyrate for *R*_g_ values (*R*_g_ represents the compactness of protein conformation), and g_energy for the potential energies between neighboring VP1 of capsomere (*E*_LJ_ and *E*_C_), g_dist for the distance between neighboring VP1 (*d*), and g_hbond for the number of hydrogen bonds existing between neighboring VP1 (*n*_H-bond_). Electrostatic potential and lipophilic potential along the molecular surfaces of the VP1 capsomere was visualized using the MOLCAD program from the SYBYL package (Tripos, http://www.tripos.com/), which is convenient for the visualization of protein surface colored according to electrostatic/lipophilic potential.

### Binding Free Energy Analysis

Binding free energy (Δ*G*_bind_) between VP1 monomers of Ag-Cap was calculated using MM-PBSA method [23],[24],[37] and the CHARMM [38],[39] (http://www.charmm-gui.org/), following the procedure reported previously [23],[24],[37]. CHARMM program is utilized because it is convenient for MM-PBSA analysis, especially its PBEQ module can be used for solving the linear Poisson-Boltzmann (PB) equation, as mentioned below. Twenty-six snapshots were extracted from the last 5 ns trajectory of each simulation at an interval of 200 ps. In these snapshots, the conformations of Ag-Cap were used for MM-PBSA analysis. Because each Ag-Cap is composed of five monomers, the average binding free energy between neighboring monomers was used for the discussion. Herein, Δ*G*_bind_ was calculated by equation 1.
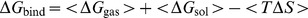
(1)

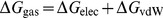
(2)

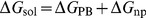
(3)

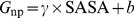
(4)


where the bracket <…> indicates an average of an energy term along the MD simulation trajectory. –*T*Δ*S* is the entropic contribution arising from changes in the degrees of freedom (translational, rotation, and vibration) of proteins, which requires large computational resource for calculation. Herein, the analysis concerned primarily per-residue electrostatic and hydrophobic contributions rather than the absolute binding free energy. Then the relative free energy was calculated without consideration of –*T*Δ*S* as that in recent literature [40],[41] and our previous work [23],[24],[37],[41]. Δ*G*_gas_ is the sum of Δ*G*_elec_ and Δ*G*_vdW_ (equation 2). The solvation energy contains the electrostatic solvation energy (Δ*G*_PB_) and the nonpolar solvation energy (Δ*G*_np_) (equation 3). Δ*G*_PB_ was calculated by solving the linear PB equation using the PBEQ module of CHARMM program. According to literature [42], the solute and solvent dielectric constants were set to 1 and 80, respectively. Δ*G*_np_ was calculated according to equation 4, where the constants *γ* and *b* were set to 0.00542 kcal/(mol·Å^2^) and 0.92 kcal/mol, respectively [43]. The solvent accessible surface area (SASA) was calculated using a water probe radius of 1.4 Å.

Free energy contribution of each residue can be divided into polar (Δ*G*_polar_) and nonpolar interactions (Δ*G*_nonpolar_) according to equation 5, and each part is the sum of two energy terms, as given in equations 6 and 7. In the following analysis, Δ*G*_polar_ is considered as the contribution by electrostatic interaction and Δ*G*_nonpolar_ as the contribution by hydrophobic interaction [24],[25],[37].
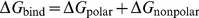
(5)

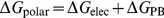
(6)

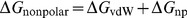
(7)


### Identification of Hot Spots

In the present study, both the residues making great contributions to the binding free energy and the residues forming important intermolecular interactions to compensate the unfavorable solvation effect were considered as the hot spots. According to literature [44], the hot spots were identified as those whose absolute value of free energy is larger than 2.5 kcal/mol. As shown in Fig. S1, each VP1 monomer has direct interaction with two neighboring VP1 monomers, leading to two binding interfaces. Herein, the two binding interfaces were labeled as A and B, respectively. In addition, due to the capsomere's symmetric structure, the calculations were performed on all five VP1 monomers, but only one VP1 was discussed to represent all five VP1 monomers. All the energy values discussed below were the average values of five VP1 molecules.

### DSC Analysis

To validate the MD simulation results, a modular Ag-Cap capsomere mutant lacking 63 C-terminal residues was expressed in *Escherichia coli* and purified as previously described [45]. Ag-Cap was buffer-exchanged into the stabilization buffer [40 mM tris, pH 8.0, 200 mM NaCl, 5 mM tris(2-carboxyethyl)phosphine (TCEP)], or assembly buffer (40 mM tris, pH 8.0, 0.5 M (NH_4_)_2_SO_4_, 1 mM CaCl_2_) during purification and centrifuged at 22 000 *g* and 4°C for 15 min. The supernatant was extracted and the protein concentration was adjusted to 1 mg/mL based on UV-Vis spectrophotometry (extinction coefficient, 1.5 L/g/cm). All buffers and samples were degassed using a MicroCal ThermoVac (GE Healthcare, NSW, Australia) prior to DSC experiments. DSC measurements were performed with a MicroCal VP-DSC microcalorimeter (GE Healthcare) with 0.497 mL reference and sample cells. Measurements were with a scan rate of 30°C/h, from 25°C to 90°C, following standard manufacturer's instructions. Data were acquired with the VPVIEWER 2000 software (version 1.4.27, MicroCal) and analyzed with ORIGIN software (Version 7.0552, OriginLab Corporation, MA, USA). The data obtained were baseline subtracted and fitted with a non-two-state model using the Levenberg-Marquardt non-linear least-square method, yielding the transition temperature (*T*_m_) and the calorimetric enthalpy (Δ*H*_m_).

The heat capacity change during the dissociation and unfolding reaction (Δ*C*_p_) was calculated from the initial and final baselines of the DSC thermogram extrapolated to *T*_m_ [46]. Using the experimentally calculated *T*_m_, Δ*H*_m_ and Δ*C*_p_, the difference in free energy between the capsomere and denatured states of VP1 (Δ*G°*) as a function of *T* was calculated using equation 8 [47].
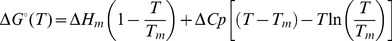
(8)


## Results and Discussion

### Electrostatic and Lipophilic Surface Potential

**Figure 1:**
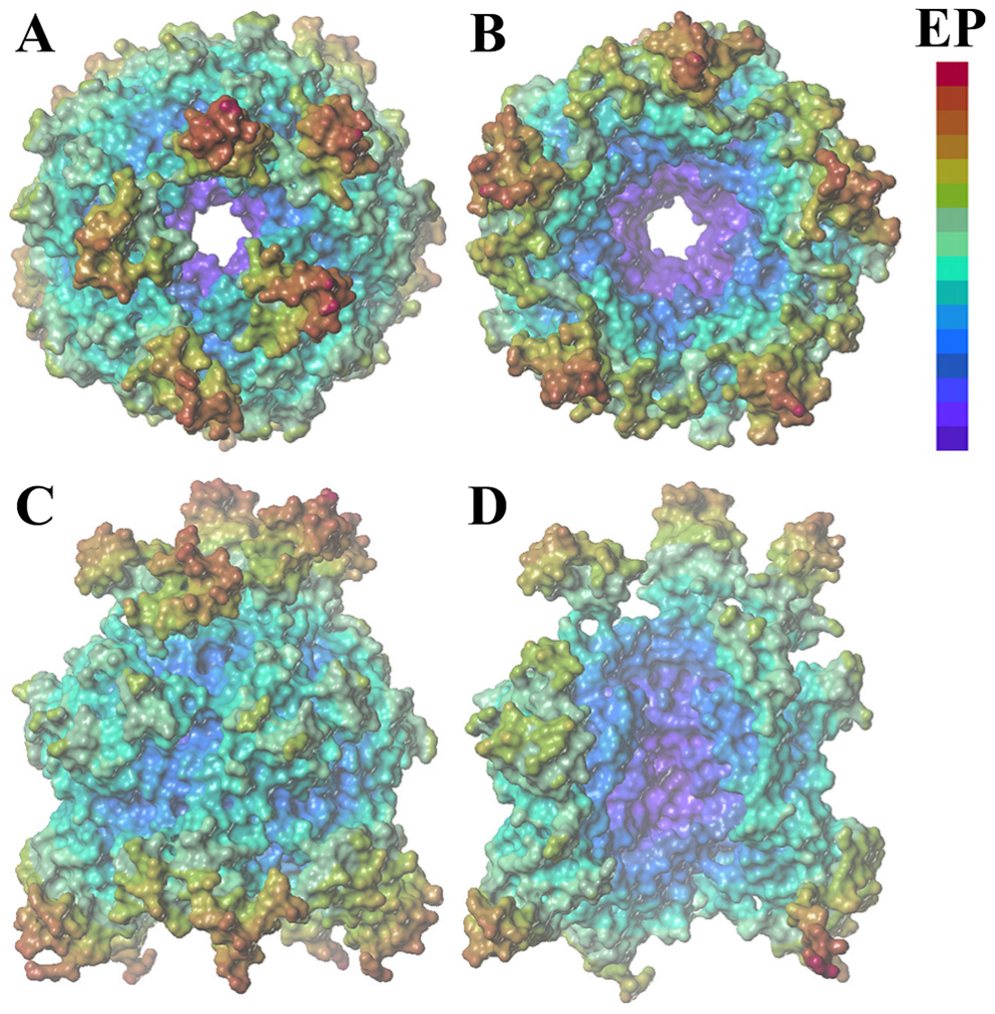
Exterior and interior surfaces of an Ag-Cap coloured according to the electrostatic potential. (A) top view, (B) bottom view, and (C) side view of the exterior surface; (D) the interior surface. In (D), only three of five VP1 monomers in a Ag-Cap are displayed to see the interior surface. Red is used for relative positive and purple for relative negative. The figures were prepared using the MOLCAD program (http://www.tripos.com/).

Exterior and interior surfaces of an Ag-Cap were coloured according to the electrostatic potential, as shown in Fig. 1. Similar to the wild-type [23], heterogeneous charge distribution is observed in Ag-Cap, including a dominant neutral exterior surface (Fig. 1C) and an almost completely negatively charged interior surface (Fig. 1D). Converged positively charged distribution is observed in the antigen fragments (Fig. 1A, 1C). Based on this simple analysis, the electrostatic repulsion between antigen fragments might be expected to cause disturbance between individual VP1 molecules and consequent destabilized structure of Cap.

**Figure 2:**
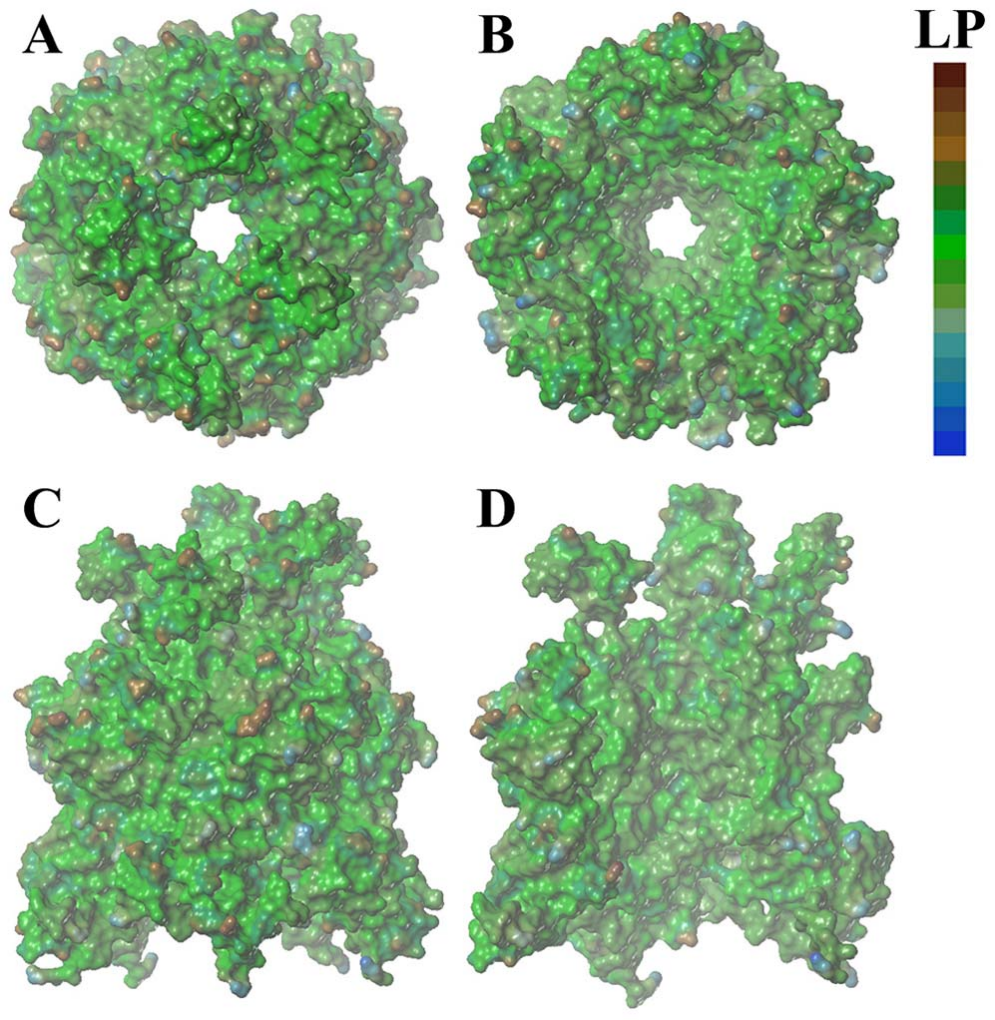
Exterior and interior surfaces of an Ag-Cap coloured according to the lipophilic potential. (A) top view, (B) bottom view, and (C) side view of the exterior surface; (D) the interior surface. Brown is used for relative hydrophobic and blue for relative hydrophilic.

Further analysis of lipophilic potential was performed to better understand the complex nature of Ag-Cap stabilization. In Fig. 2, the exterior and interior surfaces of Ag-Cap were coloured according to lipophilic potential. Similar to that for Cap, dominant neutral lipophilic potential surface is observed, with a few hydrophobic patches at the interface between neighboring VP1 molecules. The antigen is quite neutral, although a few hydrophobic points have been observed. It does not appear that the insertion of antigen modules has significant effect on the hydrophobic interaction between individual VP1 molecules, for the selected antigenic module examined here.

### Binding Free Energy Analysis

To provide further insight into the stabilization of Ag-Cap with emphasis on the effect of the insertion of antigen fragments, the binding free energies within Ag-Cap were evaluated using the MM-PBSA method. It can be seen from RMSD, *R*_g_, potential energies, distance and hydrogen bonds analysis that the Ag-Cap has been well equilibrated (Fig. S2). Noting this, the last 5 ns trajectory of each simulation was used for MM-PBSA analysis, and the effect of antigen modules on the association between neighboring VP1 was discussed.

The binding free energies of Ag-Cap in Stabilization Buffer (S3) and Assembly Buffer (S4) are listed in Table 2. Weaker binding free energies of Ag-Cap are observed in both S3 and S4 as compared with those of wild-type Cap [23]. In S3, Δ*G*_bind_ = −266 kcal/mol, which is much higher than that of Cap (Δ*G*_bind_ = −445 kcal/mol, Table 2) [23], indicates destabilization after the insertion of antigen. Δ*G*_vdW_ and Δ*G*_np_ are not seriously affected and only slightly decrease, as a result of increased hydrophobic points located in the antigen module (Fig. 2). Consequently, no significant change in Δ*G*_nonpolar_ is observed. In contrast, a large increase of Δ*G*_elec_ to −2499 kcal/mol is observed from −3516 kcal/mol in Cap, indicating unfavorable electrostatic interaction induced by the insertion of the antigen module. This finding is consistent with the electrostatic potential surface analysis in Fig. 1. The antigen fragments are predominately positively charged, leading to electrostatic repulsion. Although Δ*G*_PB_ decreases to 3232 kcal/mol, a final Δ*G*_polar_ of 733 kcal/mol is shown which is 200 kcal/mol higher than that of Cap and causes the increase of total binding free energy. Therefore, the introduction of unfavorable electrostatic repulsion is proposed as the main reason for the destabilization of Ag-Cap, and the antigen module is the main contributor.

**Table 2:** Total Binding Free Energies of Ag-Cap in Different Solutions. Unit: kcal/mol.

Protein	System	Δ*G*_vdW_	Δ*G*_np_	Δ*G*_elec_	Δ*G*_PB_	Δ*G*_nonpolar_ a	Δ*G*_polar_ b	Δ*G*_bind_ c
Ag-Cap	S3	−810(61)	−189(4)	−2499(97)	3232(136)	−999(65)	733(39)	−266(26)
	S4	−890(24)	−190(2)	−1552(120)	2406(73)	−1080(26)	855(48)	−226(74)
Cap^d^	S3	−801(27)	−178(2)	−3516(106)	4049(111)	−978(29)	533(16)	−445(39)
	S4	−774(23)	−173(4)	−2831(130)	3431(117)	−947(22)	601(16)	−346(34)

a Δ*G*_nonpolar_ = Δ*G*_vdw_+Δ*G*_np_, hydrophobic interaction energy.

b Δ*G*_polar_ = Δ*G*_elec_+Δ*G*_PB_, electrostatic interaction energy.

c Δ*G*_bind_ = Δ*G*_nonpolar_+Δ*G*_polar_.

d The data for Cap reported previously [23] were used herein as control.

Similar changes are observed in S4 but with a minor difference. Δ*G*_bind_ of Ag-Cap is −226 kcal/mol, higher than that of Cap (−346 kcal/mol, Table 2) [23]. Significant increase of Δ*G*_elec_ to −1552 kcal/mol in Ag-Cap from −2831 kcal/mol in Cap [23] is observed, leading to a higher Δ*G*_polar_ of 855 kcal/mol, confirming the unfavorable electrostatic repulsion introduced by antigen fragments. Therefore, from the results in both S3 and S4, the destabilization indicated by the increase of Δ*G*_bind_ can be attributed to the unfavorable electrostatic repulsion introduced by antigen fragments as indicated by increased Δ*G*_polar_.

Meanwhile, higher Δ*G*_bind_ of Ag-Cap in S4 is observed than that in S3, indicating the more active state in S4 favoring its self-assembly. Changes in free energy within Cap due to changes in solution conditions has been proven critically important during VLP assembly [23]. Herein, increase of Δ*G*_polar_ also confirms the significant effect of ionic strength on the association between neighboring VP1 within Ag-Cap, where electrostatic repulsion has main contribution, which is consistent with that for Cap as demonstrated previously [23]. Moreover, it should be noted that the total energetic change (Δ*G*_bind_) between S3 and S4 decreases from 99 kcal/mol for Cap [23] to 40 kcal/mol for Ag-Cap. That change of free energy value means the effect of solution conditions is reduced by the insertion of this selected antigenic module. Therefore, more careful control of the solution conditions should be provided to ensure facile self-assembly of Ag-Cap. This finding emphasizes the important role of screening for buffer conditions which can stabilize VLPs even under high thermal stress, as demonstrated using recent experimental work [48].

**Figure 3:**
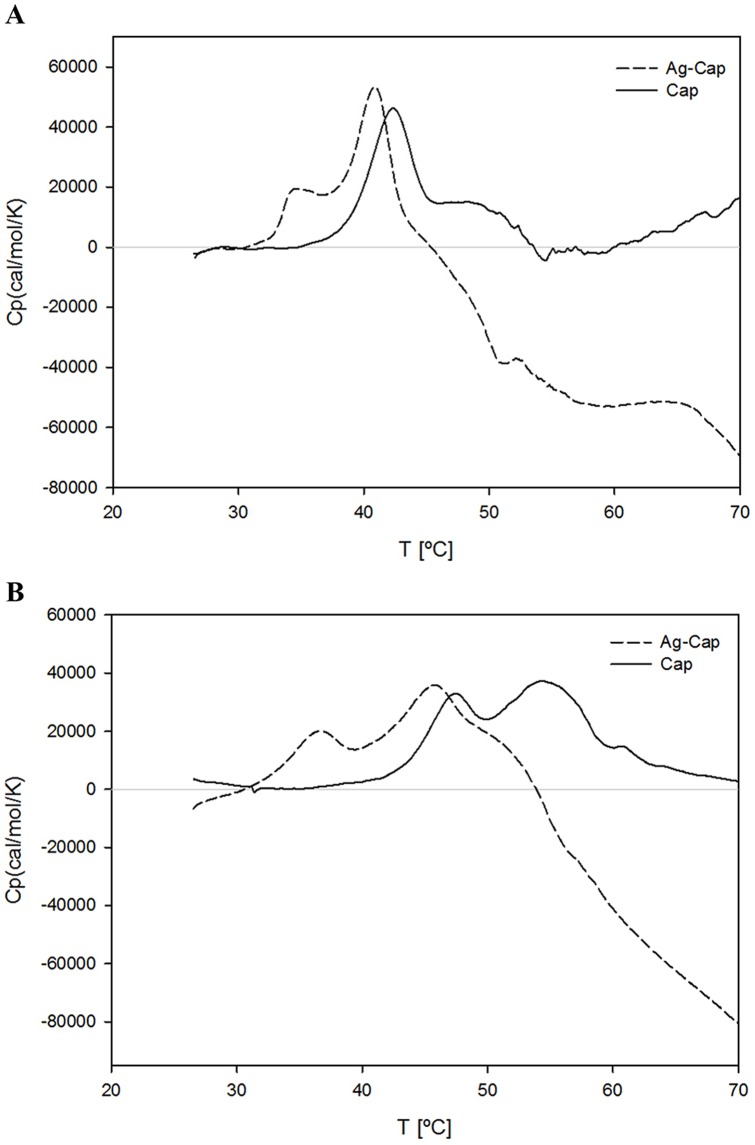
Differential scanning calorimetric thermograms of the Cap and Ag-Cap in different solution conditions. (A) Stabilization and (B) Assembly Buffers.

The data obtained from the binding free energy analysis (Table 2) was compared with experimentally-calculated free energies; DSC analysis was performed to ascertain the free energy of dissociation of modified (assembly-incompetent) Ag-Cap capsomeres [45]. Fig. 3 shows the DSC thermograms of Ag-Cap in Stabilization and Assembly Buffers, in comparison to an unmodified Cap. The thermogram in Fig. 3A is for both Cap and Ag-Cap in S3, where Ag-Cap has two endothermic events at approximately 35°C and 40°C and Cap shows a single event at the slightly higher temperature of approximately 42°C. The single endothermic event is representative of co-operative and simultaneous capsomere disassembly and VP1-monomer-unfolding under this buffer condition, as was previously observed [23]. For this reason, the Δ*G*° of dissociation at 25°C cannot be accurately calculated for Cap, as we cannot distinguish capsomere dissociation from VP1 unfolding in a single endothermic event. Ag-Cap shows in contrast two endothermic events, suggesting that the capsomere initially disassembles before the protein unfolds, thus the first event will be used to calculate thermodynamic parameters for capsomere dissociation. The higher temperature of the Cap endotherm is indicative of a more stable capsomere in this buffer condition, when compared with Ag-Cap.

The second thermogram in Fig. 3B shows Cap and Ag-Cap in S4. For each construct, two endothermic events are observed, although each event occurs at much lower temperatures for Ag-Cap in comparison to Cap. Endothermic events for Cap occur at both 46.7°C and 54.4°C and for Ag-Cap at both 36.8°C and 46.0°C. These two endotherms are representative of a dissociation/denaturation process whereby a capsomere (or assembled/aggregated capsomere) initially dissociates and then unfolds. The lower temperature of the Ag-Cap endotherm confirms less stable structure in S4 as compared with wild-type Cap. The first endotherm is therefore used for the calculation of capsomere dissociation thermodynamic parameters.

The thermodynamic parameters calculated from DSC analysis are summarized in Table 3. The effect of solution conditions on the calculated Δ*G*° of dissociation at 25°C are in agreement with the relative free energy rankings by MD simulations. Despite the inability to obtain a Δ*G*° of dissociation value for Cap in S3, due to capsomere dissociation occurring simultaneously with protein denaturation, the thermogram for Cap against Ag-Cap show a clear decrease in stability due to antigen insertion. Meanwhile, Ag-Cap was shown to have a slightly lower simulation binding energy in S3 than in S4, indicating higher stability in S3. This was also observed experimentally, with slightly higher capsomere dissociation energy in S3 in comparison to S4 (Fig. 3). It can be seen from the thermogram in S4, that Ag-Cap begins protein unfolding at a temperature lower than that at which Cap begins capsomere dissociation. This suggests that Ag-Cap is less stable in S4 than Cap.

**Table 3:** Thermodynamic Parameters from Calorimetric Scans of Cap and Ag-Cap in Different Solution Conditions.

Protein	System	*T_m_* (°C)	Δ*H_m_* (kcal mol^−1^)	Δ*Cp* (kcal mol^−1^ K^−1^)	Δ*G*° at 25°C (kcal mol^−1^)
Cap	S3	42.64±0.09	-	-	-
	S4	46.74±0.06	87.56±6.3	27.02	−124.02
Ag-Cap	S3	35.67±0.39	101.2±19	53.45	−65.1
	S4	36.82±3.1	119.5±14.5	27.97	−98.3

**Figure 4:**
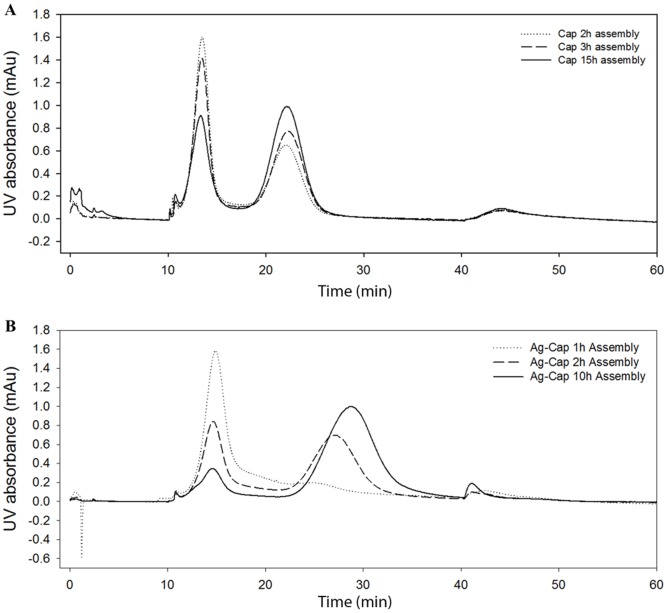
AF4 analyses showing assembly of Cap and Ag-Cap VLPs in Assembly Buffer over time.

Despite this observation of instability, experimentally we can obtain assembled Ag-Cap VLPs as characterized by AF4 in Fig. 4. Cap assembly over time can be seen in Fig. 4A, and Ag-Cap assembly over time can be seen in Fig. 4B. The results show that, as time proceeds, the first capsomere peak gets smaller while the second VLP peak gets larger, consistent with a process of VLP assembly proceeding dynamically. Importantly, despite capsomere instability in Ag-Cap, VLP assembly is still obtained for this construct. Nevertheless, we anticipate that simulation results can provide further in-depth analysis on the effects of inserting different, perhaps more destabilizing modules, on the self-assembly of VLP at the molecular level, and can provide additional insights even for this design, as discussed below.

### Free Energy Decomposition of Ag-Cap in Different Solutions

**Figure 5:**
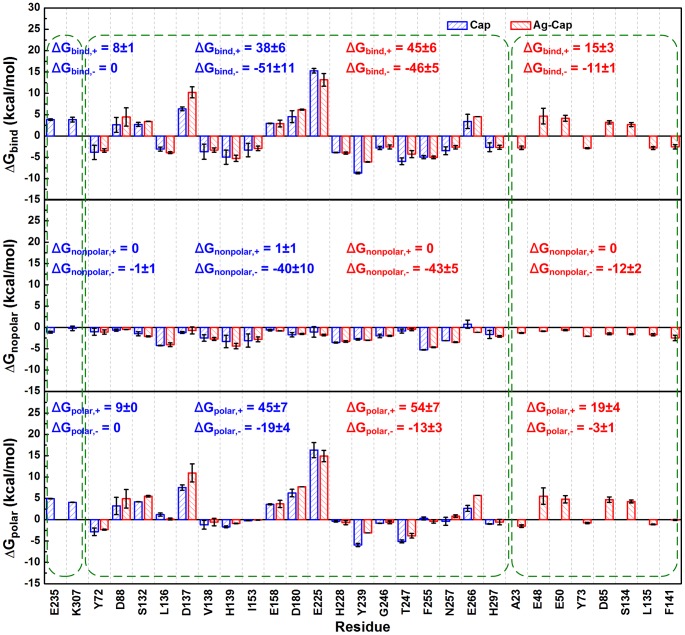
Binding free energy contribution of each residue in the Stabilization Buffer. Top panel, Δ*G*_bind_; Middle panel, Δ*G*_nonpolar_; Bottom panel, Δ*G*_polar_. The results for a Cap are compared with those for an Ag-Cap. For clarity, only the residues with the most favorable (<−2.5 kcal/mol) or unfavorable (>2.5 kcal/mol) contributions are shown. These residues are divided into three parts, i.e., residues only observed in Cap, residues observed in both Cap and Ag-Cap, and residues only observed in Ag-Cap. In each part, the residues are further divided into two clusters, including one favorable for binding and the other unfavorable for binding. The sum of the binding free energy in each cluster is calculated and provided in the figure.

For further in-depth discussion, the binding free energy contribution of each residue within an Ag-Cap in Stabilization Buffer is compared with that of Cap (Fig. 5). Herein, the residues whose absolute value of free energy is larger than 2.5 kcal/mol were identified as key residues (hot spots) and shown. Two key residues, E235 and K307 were identified in Cap but become insignificant in Ag-Cap. Both are unfavorable for binding with very high Δ*G*_bind_. The sum of unfavorable binding free energy (Δ*G*_bind,+_) is 8 kcal/mol, while that of favorable binding free energy (Δ*G*_bind,-_) is 0. Meanwhile, eight key residues are not observed in Cap but appear in Ag-Cap, including A23, E48, E50, Y73, D85, S134, L135, F141, some of which make favorable contributions for binding, e.g., A23 and L135. However, some make unfavorable contributions, e.g., E48 and E50, leading to a higher Δ*G*_bind,+_ of 15 kcal/mol as well as a lower Δ*G*_bind,-_ of −11 kcal/mol. For the key residues observed in both Cap and Ag-Cap, significant increases of both Δ*G*_bind,+_ and Δ*G*_bind,-_ are observed in Ag-Cap. Therefore, it is clear that the favorable contribution for binding is diminished, leading to a higher Δ*G*_bind_ (Table 2) and lower stabilization of Ag-Cap.

Furthermore, the free energy is decomposed into nonpolar and polar parts to evaluate the contributions from hydrophobic and electrostatic interactions, respectively. It can be seen that the key residues appearing in Ag-Cap provide much lower negative Δ*G*_nonpolar,-_ of -12 kcal/mol, but are diminished by higher Δ*G*_polar,+_ of 19 kcal/mol, indicating that the contribution of hydrophobic interaction is diminished and the electrostatic interaction is the main reason for the destabilization. With key residues both observed in Cap and Ag-Cap, significant increase is observed in both Δ*G*_polar,+_ and Δ*G*_polar,-_, confirming that the destabilized binding in Ag-Cap is mainly caused by electrostatic interaction of the antigen fragments. The contributions from each individual part are summarized in Table 4.

**Table 4:** Comparison on Free Energy Contributions of Key Residues between an unmodified capsomere and an Ag-Cap in Different Solution Conditions.

System	Unfavorable H^a^	Favorable H	Unfavorable E^b^	Favorable E	Unfavorable contribution	Favorable contribution	Dominant factor
S3	-c	↓	↑	-	↑	-	E
S4	-	-	↑	-	↑	-	E

a Hydrophobic interaction.

b Electrostatic interaction.

c ↑, ↓ and - represent enhancement, reduction, and no significant change, respectively. It should be noted that the enhancement or reduction is for the molecular interaction rather than the algebraic value of the binding free energy. For example, the increase of algebraic value of binding free energy indicates the reduction of binding and thus marked by ↓, because higher algebraic value indicates less stable binding.

**Figure 6:**
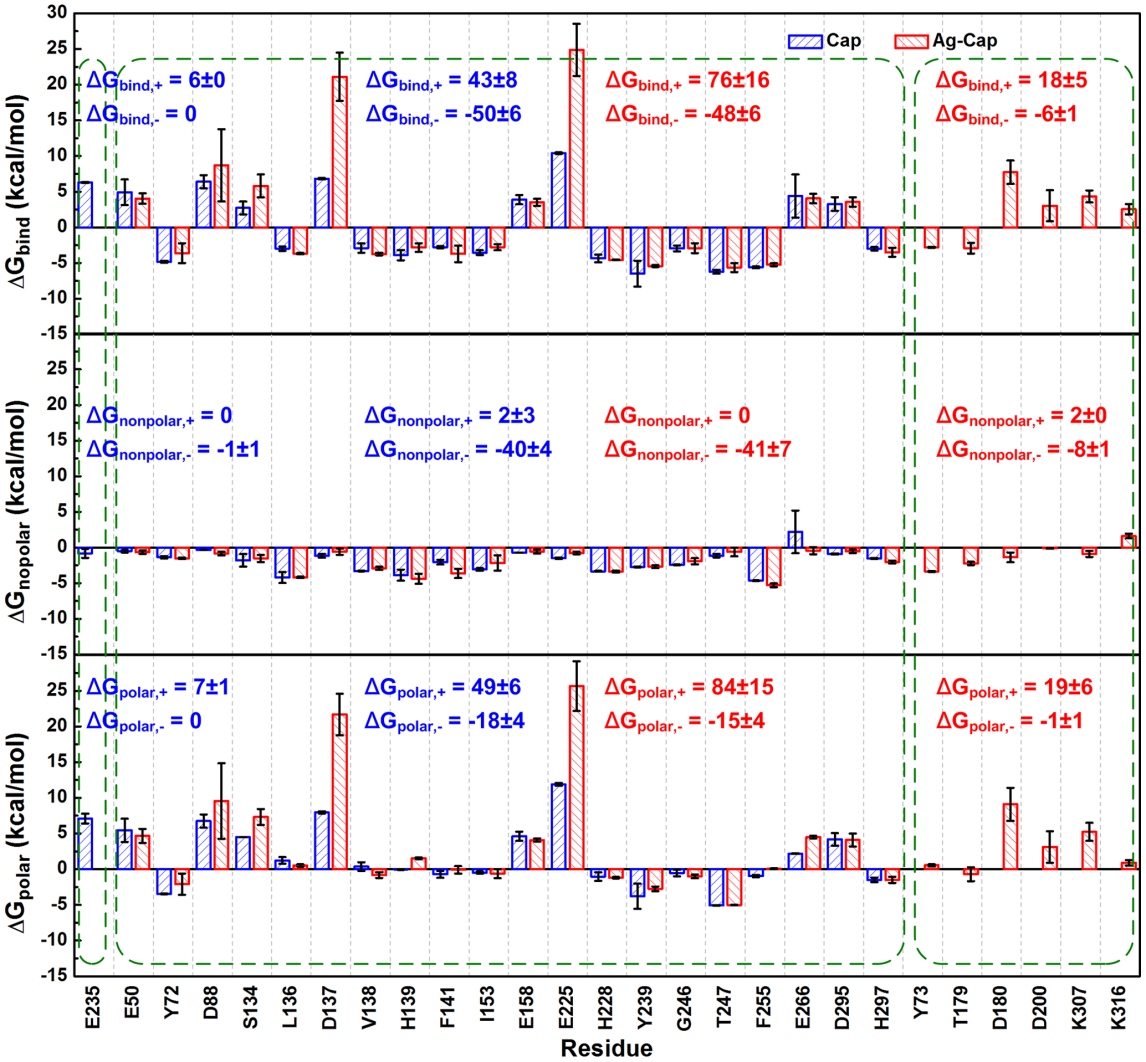
Binding free energy contribution of each residue in the Assembly Buffer. Top panel, Δ*G*_bind_; Middle panel, Δ*G*_nonpolar_; Bottom panel, Δ*G*_polar_. The results for a Cap are compared with those for an Ag-Cap. Using the same protocol in Figure 5, the most important residues are selected and the sum of the binding free energies is calculated.

The free energy decomposition in the Assembly Buffer was also performed and compared to the results in Cap. As shown in Fig. 6, 20 key residues are observed in both Cap and Ag-Cap, including eight residues favorable (E50, D88, S134, D137, E158, E225, E266, D295) and 12 residues unfavorable (Y72, L136, V138, H139, F141, I153, H228, Y239, G246, T247, F255, H297). Among these residues, a significant increase of unfavorable contribution is observed, as indicated by the increase of Δ*G*_bind,+_ from 43 to 76 kcal/mol. Moreover, one residue with Δ*G*_bind,+_ of 6 kcal/mol in Cap (E235) disappears in Ag-Cap, replaced by four residues with Δ*G*_bind,+_ of 18 kcal/mol (D180, D200, K307, K316), leading to an enhanced unfavorable contribution. This is the main reason for the increase of Δ*G*_bind_ in Table 2.

To evaluate the contributions from hydrophobic and electrostatic interactions, it is shown that nonpolar contribution (Δ*G*_nonpolar,-_) is not changed but unfavorable polar contribution (Δ*G*_polar,-_) is enhanced in Ag-Cap, confirming electrostatic interaction as the key factor (Table 2). That is, after the insertion of antigen fragments, unfavorable electrostatic interactions are enhanced, leading to the more unstable (more active) state. The changes are summarized in Table 4 in more details.

### Identification of Hot Spots

**Figure 7:**
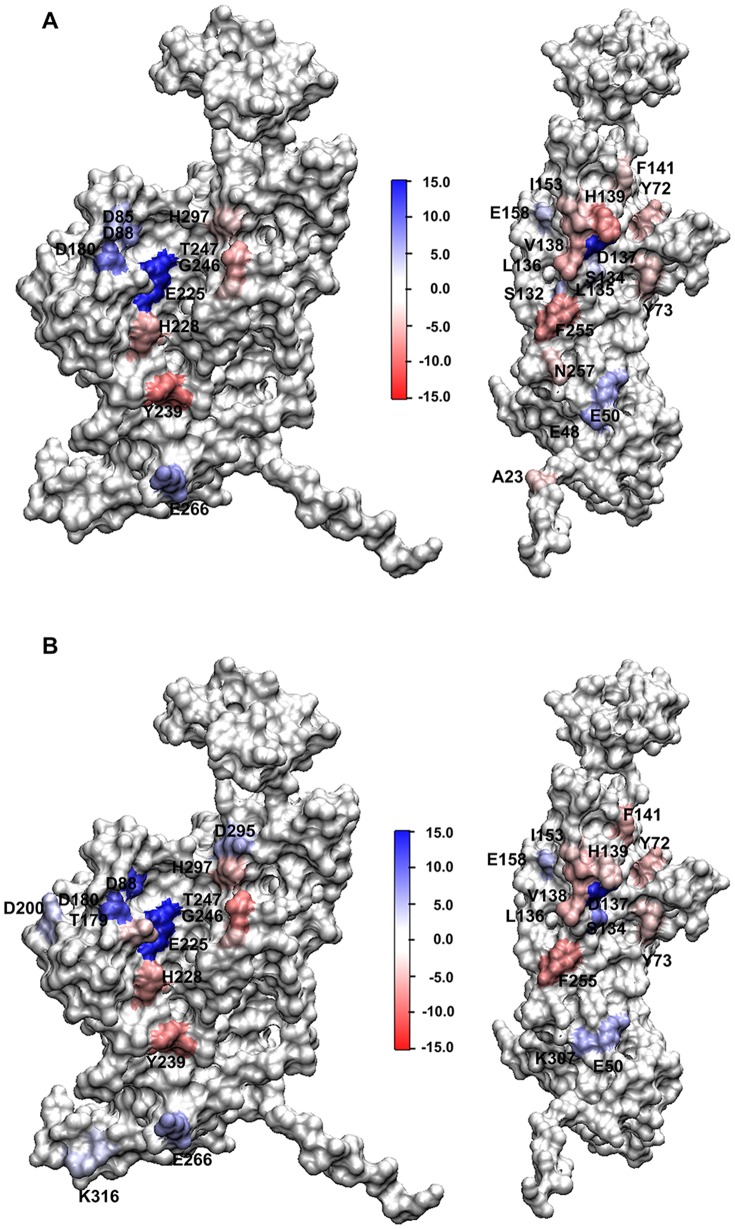
Interfaces of VP1 in Ag-Cap coloured according to the free energy contribution of each residue. (A) Stabilization and (B) Assembly Buffers. In each figure, interface A is shown on left while interface B is shown on right. For clarity, only the residues with the most favorable (<−2.5 kcal/mol) or unfavorable (>2.5 kcal/mol) contributions are labeled. Binding free energy ranges from red (most negative) to blue (most positive). The figures are prepared using the VMD software (http://www.ks.uiuc.edu/Research/vmd/).

For better understanding of the molecular interactions within Ag-Cap, the hot spots identified based on the free energy decomposition results are compared with those of Cap (Fig. 7).

In S3, 27 key residues with great contributions (Fig. 7A) are observed in Ag-Cap, including 10 key residues (D85, D88, D180, E225, H228, Y239, G246, T247, E266, and H297) at interface A and 17 key residues (A23, E48, E50, Y72, Y73, S132, S134, L135, L136, D137, V138, H139, F141, I153, E158, F255 and N257) at interface B. As compared with the interfaces in wild-type Cap [23], similar favorable patches are observed in both interfaces A and B in S3. However, the changes of unfavorable patches are complicated. Some unfavorable patches, such as E235 and K307 in Cap disappear, while some new unfavorable patches, such as E48, E50 and D85 appear in Ag-Cap. Moreover, D85 and D88 contribute to a larger unfavorable patch at interface A, while E48 and E50 form a converged unfavorable patch at interface B, leading to enhanced unfavorable contribution by electrostatic interaction, which is consistent with the results of free energy decomposition (Table 2) and provides more evidence in a direct manner.

In S4, 26 key residues with great contributions (Fig. 7B) are observed, including 13 key residues (D88, T179, D180, D200, E225, H228, Y239, G246, T247, E266, D295, H297 and K316) at interface A and 13 key residues (E50, Y72, Y73, S134, L136, D137, V138, H139, F141, I153, E158, F255 and K307) at interface B. As compared to Cap [23], more charged residues, such as D180 and D200 appear in Ag-Cap and form converged unfavorable patches, leading to enhanced unfavorable electrostatic interaction (Tables 2 and 4).

## Conclusions

MD simulations coupled with MM-PBSA analysis and experimental calorimetry analysis were used to examine the molecular interactions within an Ag-Cap with emphasis on the change of conformational stability of capsomeres induced by inserting an antigen module. Weaker binding free energy is observed after the insertion of an influenza antigen module, and a more active state is observed in Assembly Buffer as compared with that in Stabilization Buffer. Further in-depth analysis based on free energy decomposition indicates that unfavorable electrostatic repulsion induced by the antigen module is the main reason for the destabilization of Ag-Cap. Despite this energetic destabilization, experiments reveal the successful formation of VLPs even from the activated state, suggesting that some level of activation is acceptable from a practical sense, but also that a threshold destabilization point likely exists, which will be design-dependent. This work also shows that the influence of solution conditions is reduced by insertion of the chosen antigen module. This finding highlights the potential to screen for solution conditions that favor good self-assembly of Ag-Cap, which we anticipate will be highly design-specific. This work has provided molecular insights into the conformational stability of capsomeres and the influence of antigen insertion, which would be helpful for engineering the surface of modular VLPs to enhance immunogenicity of selected antigens and the rational engineering of novel modular VLP-based vaccines. Future designs of modular VLP vaccines can be facilitated by the analysis of energetic properties of the capsomeres, and the prediction of favorable and/or unfavorable forces being introduced. Thus designs can be tailored to improve energetics, whilst maintaining the desired immunological properties of the module. In addition, solution conditions can be optimized to suit the modular VLPs to cater for the changes in surface properties due to module insertion. Overall, this work will provide insight into the biophysical effects of modularization and improve the design process for modular VLP vaccines.

## Supporting Information

Figure S1 **Conformation of Ag-Cap colored by chain.** (A) top view and (B) side view. The antigen fragments (GCN4-H190-GCN4) are shown in yellow. The figures were prepared using the visual molecular dynamics (VMD) software (http://www.ks.uiuc.edu/Research/vmd/).
(TIF)

Figure S2 **Dynamic behaviors of Ag-Cap in different solution conditions.** The time courses of RMSD and *R*_g_ values (A), potential energies of Ag-Cap (B), and the distance and the number of hydrogen bonds between neighboring VP1 (C) are shown.
(TIF)

Table S1 **Atom Numbers in Each Simulation System.**
(DOC)
