# Supplementary figures and images for: Energetic Changes Caused by Antigenic Module Insertion in a Virus-Like Particle Revealed by Experiment and Molecular Dynamics Simulations
Source: PLoS One. 2014 Sep 12;9(9):e107313. doi: 10.1371/journal.pone.0107313 (PMC4162605; doi:10.1371/journal.pone.0107313)

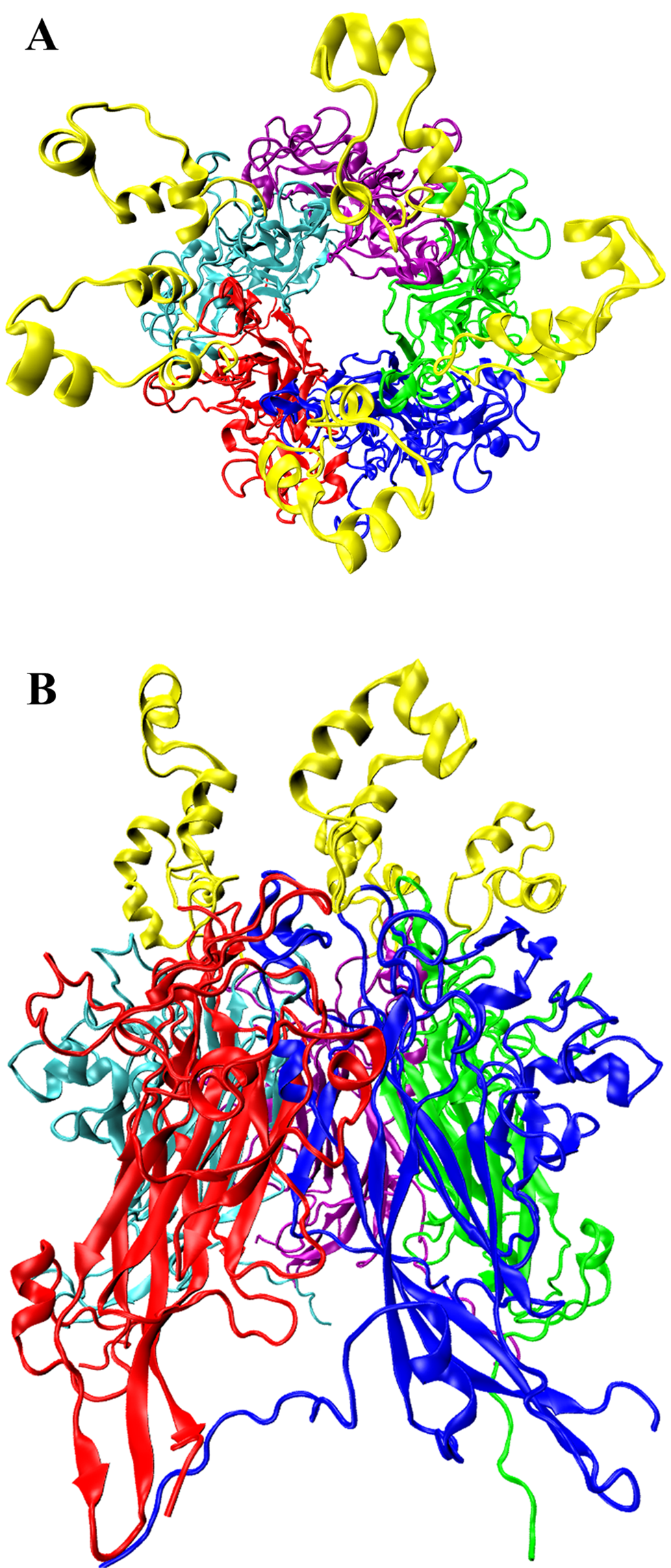

Supplement: Figure S1 — Conformation of Ag-Cap colored by chain. (A) top view and (B) side view. The antigen fragments (GCN4-H190-GCN4) are shown in yellow. The figures were prepared using the visual molecular dynamics (VMD) software (http://www.ks.uiuc.edu/Research/vmd/). (TIF) [file pone.0107313.s001.tif]

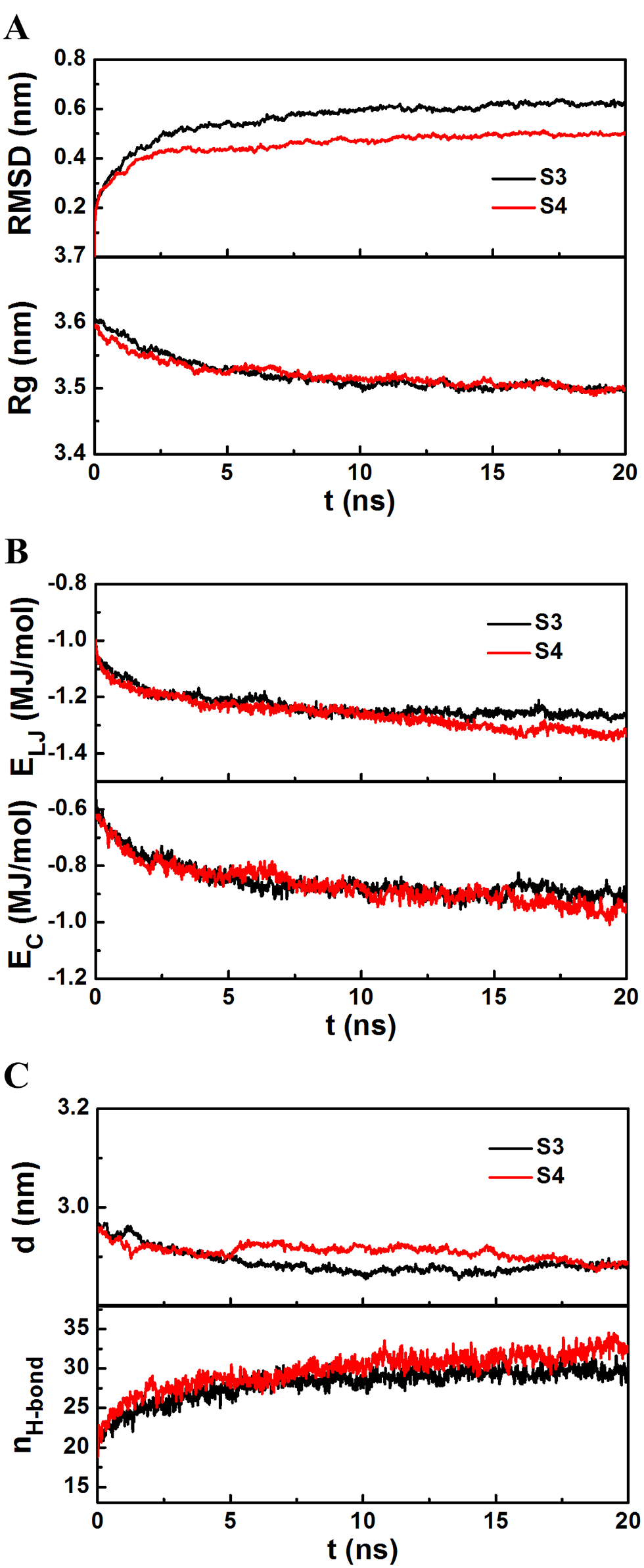

Supplement: Figure S2 — Dynamic behaviors of Ag-Cap in different solution conditions. The time courses of RMSD and R g values (A), potential energies of Ag-Cap (B), and the distance and the number of hydrogen bonds between neighboring VP1 (C) are shown. (TIF) [file pone.0107313.s002.tif]
