# Supplementary material for: Energetic Changes Caused by Antigenic Module Insertion in a Virus-Like Particle Revealed by Experiment and Molecular Dynamics Simulations
Source: PLoS One. 2014 Sep 12;9(9):e107313. doi: 10.1371/journal.pone.0107313 (PMC4162605; doi:10.1371/journal.pone.0107313)
Supplement: Table S1 — Atom Numbers in Each Simulation System. (DOC) [file pone.0107313.s003.doc]

**Table S1.** Atom Numbers in Each Simulation System.

| System | Number of Na^+^ | Number of Cl^-^ | Number of NH_4_^+^ | Number of SO_4_^2-^ | Number of Ca^2+^ | Number of water |
| --- | --- | --- | --- | --- | --- | --- |
| S3 | 258 | 233 | 0 | 0 | 0 | 64509 |
| S4 | 25 | 2 | 1322 | 661 | 1 | 60208 |
